# PPARα: Linking Cardiac Metabolism to Therapeutic Opportunities in Cardiovascular Diseases

**DOI:** 10.3390/cells15100940

**Published:** 2026-05-20

**Authors:** Maxime Roes, Claude Libert, Jolien Vandewalle

**Affiliations:** 1 Center for Inflammation Research, Vlaams Instituut voor Biotechnologie (VIB), 9052 Ghent, Belgium; maxime.roes@irc.vib-ugent.be (M.R.); claude.libert@irc.vib-ugent.be (C.L.); 2 Department of Biomedical Molecular Biology, Ghent University, 9052 Ghent, Belgium

**Keywords:** PPARα, heart, sepsis, ischemia/reperfusion, diabetes

## Abstract

Peroxisome proliferator-activated receptor alpha (PPARα) is a key transcriptional regulator of lipid metabolism, highly expressed in metabolically active organs such as the heart. In cardiomyocytes, where approximately 70% of energy is derived from fatty acid oxidation, PPARα plays a central role in maintaining metabolic homeostasis. Moreover, the transcription factor is implicated in postnatal maturation of the heart and immune modulation. Dysregulation of PPARα signaling has profound consequences for cardiac energy balance, particularly under stress conditions. Accordingly, its role has been extensively investigated in cardiovascular diseases, including ischemia/reperfusion, diabetic cardiomyopathy and sepsis-induced cardiomyopathy. Upon ischemia/reperfusion and sepsis, cardiac PPARα expression is typically downregulated, contributing to impaired fatty acid breakdown and reduced metabolic flexibility. In contrast, diabetic cardiomyopathy is characterized by sustained PPARα activation, promoting excessive fatty acid oxidation, lipid accumulation and lipotoxicity. These context-dependent effects highlight a complex role of PPARα in cardiac diseases. PPARα has emerged as a promising therapeutic target, as its modulation can alleviate cardiac injury in preclinical models. However, further research is required to validate its efficacy in human disease, improve cardiomyocyte-specific targeting strategies to minimize systemic side effects, and better define optimal timing of intervention, as inappropriate or prolonged modulation may lead to detrimental outcomes.

## 1. Introduction

Cardiovascular diseases remain the leading cause of death worldwide and represent a major global health burden. With the aging population and the increasing prevalence of metabolic disorders, the incidence of cardiovascular events is expected to rise further in the coming decades [1,2]. Consequently, considerable efforts are focused on improving our understanding of the molecular mechanisms underlying cardiovascular disease development and progression.

The heart is one of the highest consumers of adenosine triphosphate (ATP) in the body, ranking second after the brain. Since the heart has a limited storage capacity for ATP, it requires a continuous supply to sustain contractile function. Consequently, cardiac metabolism has become an important area of investigation in both physiological and pathological conditions. In the healthy adult heart, approximately 75% of cardiac ATP is produced through the mitochondrial β-oxidation of fatty acids, while additional substrates such as glucose, lactate, ketone bodies and amino acids contribute to a lesser extent. Notably, cardiomyocytes exhibit remarkable metabolic flexibility, enabling dynamic shifts between substrates in response to changes in energetic demand and substrate availability [3,4].

Peroxisome proliferator-activated receptors (PPARs), members of the nuclear hormone receptor superfamily, are key transcription factors regulating this metabolic flexibility. The PPAR family consists of three isoforms: PPARα, PPARδ and PPARγ, each displaying distinct tissue expression patterns and metabolic functions. PPARα is highly expressed in tissues with high rates of fatty acid oxidation (FAO), such as liver, heart and skeletal muscle. In contrast, PPARγ is predominantly expressed in adipose tissue, while PPARδ is more ubiquitously expressed across different tissues [5].

Because of the prominent role of PPARα in regulating cardiac lipid metabolism and energy homeostasis, this family member has been extensively studied in the context of cardiovascular diseases. Dysregulation of PPARα signaling has been observed in multiple cardiovascular conditions, including ischemia and reperfusion injury, diabetic cardiomyopathy and sepsis-induced cardiomyopathy [6]. As a result, this transcription factor has emerged as an important molecular link between metabolic remodeling and cardiac dysfunction and represents a promising therapeutic target in cardiovascular disease.

In this review, we discuss the role of PPARα in cardiac metabolism and highlight its involvement in various cardiovascular pathologies. Particular emphasis is placed on ischemia/reperfusion injury, where PPARα is downregulated, diabetic cardiomyopathy, in which PPARα is upregulated, and sepsis-induced cardiomyopathy, characterized by an initial activation of PPARα followed by subsequent downregulation. These conditions are discussed to illustrate the pathological consequences of both excessive and insufficient PPARα expression. Additionally, its potential as a therapeutic target is explored.

## 2. Structure and Activation of PPARα

**Figure 1:**
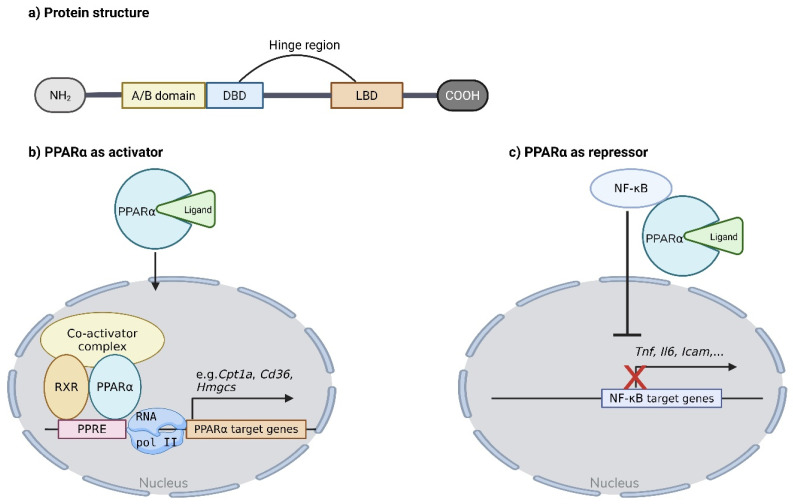
Structure and activation mechanism of PPARα. (**a**) The PPARα protein contains an A/B domain at the N-terminus, followed by the DNA-binding domain (DBD). This domain is connected to the ligand-binding domain (LBD) at the C-terminus through a hinge region. (**b**) Upon ligand binding in the LBD, PPARα translocates to the nucleus and forms a heterodimer with retinoid X receptor (RXR). The complex binds to PPAR response elements (PPREs) in the promoter regions of target genes, leading to transcriptional activation. (**c**) PPARα can also act as a repressor via direct protein–protein interactions. Upon activation, it binds to other transcription factors, for example, NF-kB, and inhibits translocation to the nucleus, preventing NF-κB target gene expression. Created in BioRender. Roes, M. (2026) https://BioRender.com/exs4bzi.

Like other nuclear receptors, PPARα is a ligand-activated transcription factor with a highly conserved modular domain structure. It exhibits high homology between species, with up to 90% sequence identity between humans and mice [7]. At the N-terminus, the A/B domain contains an activation function-1 (AF-1) region, which mediates ligand-independent transcriptional activation. This activity is relatively rare and is achieved by phosphorylation via MAPK signaling [8]. Next, the DNA-binding domain (DBD), including two zinc finger motifs, enables PPARα to bind specific DNA sequences, known as peroxisome proliferator response elements (PPREs). These elements are located upstream or within the promoters of target genes and are defined by repeats of the six-base sequence AGGTCA, separated by a single nucleotide [8,9]. Following the DBD, the hinge region connects the DBD with the ligand-binding domain (LBD) and provides conformational flexibility, facilitating interactions with co-regulators and DNA. In the absence of a ligand, the hinge region facilitates interaction with co-repressors, such as Nuclear receptor corepressor (NCoR). This region can also undergo post-translational modifications, which may modulate receptor activity [7,8]. Finally, the LDB contains the specific ligand binding pocket (LBP) that mediates ligand recognition and binding. At the C-terminus, the activation function 2 (AF-2) motif is present, which undergoes conformational stabilization upon ligand binding and recruits co-activators to initiate transcription (Figure 1a) [7,10,11].

Activation of PPARα is initiated by a ligand binding to the LBD (Figure 1b). Ligands can be of endogenous origin, including fatty acids, eicosanoids and conjugated linoleic acid, as well as synthetic origin, such as fibrates. Upon binding, PPARα translocates to the nucleus, where it heterodimerizes with the retinoid X receptor (RXR). This complex binds to PPREs in the promoter region of target genes. Ligand binding induces conformational changes in the PPARα-RXR complex, resulting in the release of co-repressors and the recruitment of co-activators. Some co-activators, such as CREB-binding protein (CBP) and steroid receptor coactivator 1 (SRC-1), possess intrinsic histone-modifying activity, whereas others, such as peroxisome proliferator-activated receptor gamma coactivator 1 alpha (PGC1α), act by recruiting additional histone-modifying enzymes, activating other transcription factors or facilitating assembly of the transcriptional machinery. Collectively, these processes remodel chromatin to increase DNA accessibility, thereby promoting target gene expression [12,13].

In addition to its role as a transcriptional activator, PPARα can also act as a transcriptional repressor (Figure 1c). The best established mechanism is through protein–protein interactions, whereby PPARα interacts with other transcription factors and suppresses transcription of downstream target genes [14]. PPARα can also induce the expression of corepressor CBFA2T3, which modulates lipid metabolism genes [15]. Finally, post-translational modifications such as sumoylation can influence PPARα behavior. Sumoylation of the LBD of PPARα promotes interaction with GA-binding protein (GABP) and recruitment of histone deacetylases and methylases, leading to methylation of activation sites and repression of target genes [16].

## 3. PPARα Is a Crucial Transcription Factor for Heart Metabolism, Maturation and Immunity

**Figure 2:**
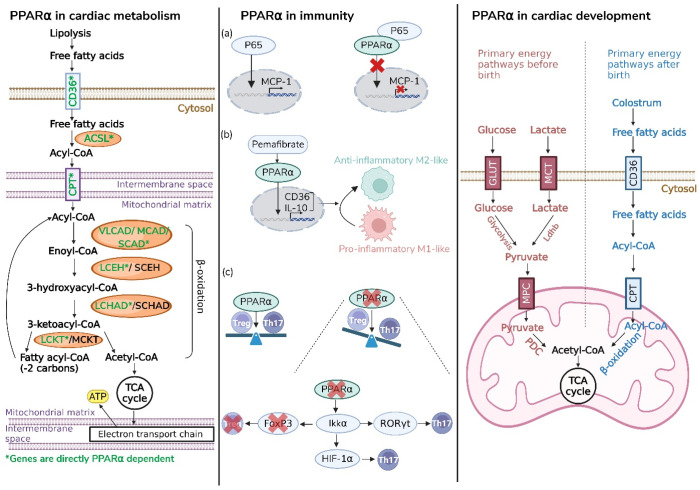
Overview of PPARα functions in the heart. **Left panel**: Upon activation of PPARα, lipolysis in white adipose tissue is stimulated, resulting in the release of free fatty acids (FFA) into the bloodstream. FFA is transported into cardiomyocytes via Cluster of Differentiation 36 (CD36). In the cytosol, FFA are converted to acyl-coA by acyl-coA synthetase (ACSL) and transported into the mitochondria via carnitine palmitoyltransferase (CPT). Within the mitochondria, acyl-coA undergoes β-oxidation, generating acetyl-coA, which enters the tricarboxylic cycle (TCA). This process produces proton-rich metabolites that fuel the electron transport chain and ultimately generate adenosine triphosphate (ATP). Abbreviations: VLCAD: very long-chain acyl-coA dehydrogenase; MCAD: medium-chain acyl-coA dehydrogenase; SCAD: short-chain acyl-coA dehydrogenase; LCEH: long-chain enoyl-CoA hydratase; SCEH: short-chain enoyl-CoA hydratase; LCHAD: long-chain 3-hydroxyacyl-CoA dehydrogenase; SCHAD: short-chain 3-hydroxyacyl-CoA dehydrogenase; LCKT: long-Chain 3-ketoacyl-CoA thiolase; MCKT: medium-chain 3-ketoacyl-CoA thiolase. **Middle panel**: PPARα regulates inflammatory signaling through multiple mechanisms. (**a**) PPARα suppresses inflammatory signaling via transrepression by interacting with the p65 subunit of NF-κB, thereby limiting its nuclear translocation and reducing the transcription of pro-inflammatory genes such as monocyte chemoattractant protein 1 (MCP-1). (**b**) PPARα also modulates macrophage polarization. Upon activation by pemafibrate, PPARα translocates to the nucleus and induces target genes, including CD36 and interleukin-10 (IL-10), promoting an anti-inflammatory macrophage phenotype. (**c**) PPARα further contributes to the balance between T helper 17 cells (Th17) and regulatory T cells (Tregs). In the absence of PPARα, increased expression of Inhibitor of Nuclear Factor kappa-B Kinase subunit alpha (IKKα) enhances activation of Retinoic acid-related orphan receptor gamma t (RORyt) and hypoxia-inducible factor 1 alpha (HIF-1a), promoting Th17 differentiation. IKKα also promotes Forkhead box P3 (FoxP3) degradation, reducing Treg stability. **Right panel**: Cardiac energy metabolism changes during development. During the perinatal period, the heart primarily relies on glucose and lactate metabolism and exhibits low PPARα expression levels (red). After birth, increased FFA availability from colostrum and elevated PPARα expression drive a metabolic shift towards fatty acid oxidation (FAO) (blue). Created in BioRender. Roes, M. (2026) https://BioRender.com/1cjff1g.

The role of PPARα is extensively studied in the cardiovascular system due to its importance after birth. In the heart, PPARα acts as a key regulator of cardiac metabolism and maintenance of cardiac identity, while also contributing to additional processes such as inflammatory and immune responses. The following paragraphs will discuss these functions in greater detail. An overview of these functions is also given in Figure 2.

### 3.1. PPARα as Master Regulator of Cardiac Fatty Acid Metabolism

One of the principal functions of PPARα in the heart is the regulation of fatty acid metabolism. It exerts this role by coordinating the expression of a broad network of genes involved in fatty acid uptake, intracellular transport and mitochondrial oxidation (Figure 2, left panel) [17,18,19]. Under normal physiological conditions, cardiac energy supply can be maintained in the absence of PPARα, as metabolic flexibility enables compensation through alternative substrate pathways. This is supported by studies in PPARα-deficient mice, which are viable but exhibit subtle metabolic alterations, including reduced expression of FAO enzymes and a shift towards carbohydrate/lactate utilization for ATP production, accompanied by mild cardiac fibrosis [20,21,22,23,24]. More recently, cardiomyocyte-specific inducible PPARα knockout models have been developed. Comparable to the PPARα-deficient mice, these mice exhibit no pathological changes in the heart and have minor alterations, such as reduced expression of FAO genes under stress-free conditions [19]. However, a sustained shift toward increased reliance on glucose metabolism has been associated with cardiac dysfunction, as demonstrated in transgenic mice with cardiac-specific overexpression of glucose transporter type 1 (GLUT1), which exhibit reduced metabolic flexibility and impaired cardiac performance [25].

During cardiac stress, such as conditions of increased workload or disease, the importance of PPARα-driven metabolic flexibility becomes more evident. PPARα-deficient mice develop ventricular dysfunction, which has been attributed to an impaired ability to upregulate β-oxidation, resulting in insufficient energy supply [26]. In cardiomyocyte-specific inducible PPARα knockout models under stress conditions, loss of cardiac PPARα exacerbates cardiac hypertrophy and fibrosis, diminishes ATP production and impairs metabolic flexibility, ultimately leading to compromised heart function [27]. Together, these findings underscore PPARα as a central regulator of cardiac energy homeostasis and essential determinant of the heart’s ability to adapt to metabolic stress.

### 3.2. PPARα as Regulator of Postnatal Cardiac Maturation

Notably, the regulatory role of PPARα is also critical in early postnatal life. During fetal development, cardiac PPARα expression is low and the heart predominantly relies on glucose and lactate as energy substrates. In the perinatal period, exposure to oxygen and the intake of lipid-rich colostrum lead to a marked increase in circulating fatty acids, which in turn activate PPARα signaling (Figure 2, right panel). This metabolic transition from glucose to fatty acid oxidation is essential for functional and structural maturation of the heart, including cardiomyocyte maturation processes such as cell-cycle arrest, binucleation and hypertrophic growth [28]. This concept has been further supported by studies in Hmgcs2-knockout mice. Hmgcs2 is a key enzyme in ketogenesis and a PPARα-dependent target gene. Hmgcs2-knockout mice exhibit partial early postnatal lethality, and surviving animals display impaired cardiac function, underscoring the importance of lipid-dependent metabolic adaptation during this developmental window [29,30].

### 3.3. PPARα as Anti-Inflammatory Modulator

Next to its role in fatty acid metabolism, PPARα also functions as an important anti-inflammatory regulator modulating both innate and adaptive immune responses (Figure 2, middle panel). Mechanistically, PPARα suppresses inflammatory signaling via transrepression. Studies in aortic explants from PPARα-deficient mice demonstrated enhanced inflammatory activation, indicating that PPARα directly interferes with pro-inflammatory transcription factors such as Activator protein 1 (AP-1), thereby limiting downstream induction of cytokines, including interleukin 6 (IL-6) [14]. In addition, PPARα physically interacts with the p65 subunit of NF-kB, leading to repression of inflammatory mediators such as monocyte chemoattractant protein-1 (MCP-1). This anti-inflammatory effect is further enhanced by Sirtuin 1 (*Sirt1*), which promotes PPARα transcriptional activity, as studied in neonatal cardiomyocytes in vitro [31]. Beyond transcriptional repression, PPARα can modulate the immune system by interfering with macrophage polarization. In models of peritoneal inflammation, activation of PPARα with pemafibrate reduced tissue macrophage accumulation and decreased pro-inflammatory cytokine levels such as tumor necrosis factor alpha (TNF-α), while control-treated mice exhibited increased macrophage infiltration. Pharmacological activation of PPARα promoted an anti-inflammatory macrophage phenotype characterized by reduced NF-κB signaling and lower expression of inflammatory markers [32,33]. PPARα also modulates adaptive immunity by influencing T-cell differentiation. Loss of PPARα enhances the differentiation of Th17 cells from naïve CD4+ T cells, thereby disturbing the balance between Th17 cells and regulatory T cells (Tregs). Under physiological conditions, PPARα limits Th17 development by recruiting co-repressors to Retinoic acid-related orphan receptor gamma t (RORyt)-dependent target genes, contributing to immune tolerance and protection from autoimmune dysregulation [34].

Together, these findings position PPARα as a central regulator of cardiac physiology throughout life, integrating postnatal cardiac maturation, metabolic control and immune modulation. By coordinating these pathways, PPARα ensures cardiac metabolic flexibility and functional stability. Disruption of this tightly regulated network may therefore predispose the heart to maladaptive remodeling and contribute to the development of pathological conditions.

## 4. PPARα Is Implicated in Several Pathological Heart Conditions

**Figure 3:**
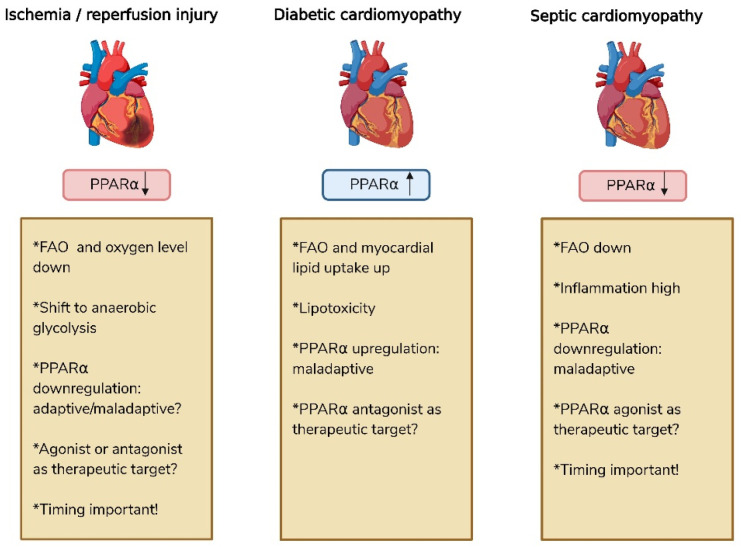
Differential expression of PPARα and consequences in several cardiac diseases. PPARα expression is increased in diabetic cardiomyopathy(DCM), but reduced in ischemia/reperfusion injury (I/R) and sepsis-induced cardiomyopathy (SICM). Studies in DCM and SICM suggest that PPARα represents a potential therapeutic target for improving metabolic dysregulation. In contrast, the role of PPARα in I/R remains controversial, as both beneficial and detrimental effects of PPARα modulation have been reported. Created in BioRender. Roes, M. (2026) https://BioRender.com/k8jlu2s.

As mentioned, PPARα plays a central role in multiple aspects of cardiac physiology, including metabolism, immunity and postnatal maturation. In recent years, research has increasingly focused on its involvement in cardiovascular diseases (CVDs). However, because CVDs encompass a heterogeneous group of pathologies, the function and behavior of PPARα may vary depending on the specific pathological context. The following sections focus on three representative modes of PPARα regulation in cardiovascular pathology (i.e., downregulated, upregulated, and dynamically regulated), illustrated by ischemia/reperfusion injury, diabetic cardiomyopathy, and sepsis-induced cardiac dysfunction as CVD, respectively (Figure 3). To study the consequences of these variations for cardiac function, numerous studies have employed pharmacological activation or inhibition of PPARα, as well as genetic mouse models, to investigate its functional significance.

### 4.1. Reduced PPARα Signaling in Cardiovascular Pathology

Several CVDs are associated with reduced PPARα signaling, including ischemia/reperfusion injury (I/R), cardiac hypertrophy and heart failure. I/R injury represents an early and acute form of metabolic stress in which alterations of PPARα signaling directly influence cardiomyocyte survival and cardiac remodeling. In contrast, heart failure and cardiac hypertrophy are chronic conditions characterized by progressive metabolic remodeling. Moreover, heart failure is a heterogeneous chronic end-stage syndrome with multiple potential underlying causes. Here, the consequences and therapeutic advancements of PPARα in I/R are discussed in detail as an illustrative example, as it nicely shows the stage-dependent role of PPARα downregulation and direct consequences, while the other two conditions are more chronic. Recent literature (<5 years) about heart failure will be summarized in Table 1 to provide a concise overview of the field. In Table 2, this is done for cardiac hypertrophy.

Myocardial ischemia is characterized by reduced or absent blood flow due to coronary artery occlusion, leading to acute ischemic heart disease. The prevalence of myocardial ischemia reaches up to 9.5% in individuals over 60 years and accounts for approximately 16% of global mortality, making it the leading contributor to cardiovascular-related deaths [51,52]. Current therapeutic strategies focus on the rapid restoration of coronary blood flow, a process known as reperfusion, typically achieved through thrombolytic therapy or, when available, percutaneous coronary intervention with stent placement. Although reperfusion has significantly reduced ischemia-related mortality, it paradoxically induces additional myocardial injury through mechanisms such as reactive oxygen species (ROS) generation, calcium overload, inflammation and metabolic changes [53,54]. Despite successful reperfusion, 30-day mortality is estimated at 5%, and one-year post-infarction mortality remains approximately 10%, with even higher numbers in older patients or those with delayed reperfusion, reflecting both the initial ischemic damage and the contribution of reperfusion injury [55,56].

Among the metabolic alterations occurring during myocardial I/R, lipid metabolism is particularly affected. Myocardial PPARα expression is downregulated following ischemia and remains suppressed during early reperfusion, resulting in decreased FAO and a compensatory shift towards anaerobic glycolysis for energy production [54,57]. This early downregulation may represent a protective adaptation to oxygen scarcity, as FAO is highly oxygen-dependent, enabling the heart to maintain ATP levels and preserve contractility despite limited oxygen availability. During later reperfusion, PPARα levels partially recover depending on the severity of the injury and ischemia duration, yet remain lower than in healthy controls [57]. This persistent downregulation is most likely maladaptive, driven by ROS bursts and mitochondrial damage, leading to impaired metabolic flexibility and progression to heart failure. Consequently, therapeutic strategies targeting PPARα activation after reperfusion have been investigated. However, their efficacy remains controversial, with both beneficial and detrimental outcomes reported.

Multiple studies support a protective role for PPARα activation in I/R. Mo et al. (2016) established a rat model of cardiac I/R and showed that pretreatment with fenofibrate protected the myocardium against injury and restored the reduced PPARα mRNA expression levels [57]. In addition, infarct size and cardiomyocyte death were significantly reduced, while mitochondrial damage and ROS production were attenuated. Similarly, other studies have demonstrated that pretreatment with a PPARα activator decreases myocardial infarct size and nearly restores cardiac function to control levels. Mechanistically, these protective effects have been attributed to inhibition of ferroptosis, an iron-dependent form of cell death driven by lipid peroxidation, and mitigation of mitochondrial injury, ultimately leading to improved outcomes after I/R [57,58]. In addition, the effect of PPARα on other cell types in the heart can contribute to the protective role of PPARα in the heart.

In contrast, other evidence suggests that PPARα activation may exacerbate I/R injury. To investigate this, murine models of cardiomyocyte-specific PPARα overexpression and whole-body PPARα knockout mice have been generated. In mice with cardiomyocyte-specific PPARα overexpression, FAO rates were increased and correlated with impaired recovery of cardiac power after reperfusion compared to wild-type littermates. This points to a negative effect of PPARα on cardiomyocytes during I/R injury. Conversely, whole-body PPARα-deficient mice displayed significantly improved recovery of cardiac power following I/R injury compared to control subjects, suggesting that loss of PPARα is beneficial in this context [58]. Thus, although cardiac PPARα overexpression is sufficient to impair recovery, the beneficial effects of whole-body PPARα deficiency may reflect contributions from non-cardiac tissues. Consistent with these findings, in a model of repetitive I/R without myocardial infarction, mice with cardiac-specific overexpression developed easier microinfarctions, which were accompanied by worse left ventricular dysfunction, supporting the detrimental effect of cardiac PPARα during I/R [59]. Similarly, pharmacological activation of PPARα using WY-14643 produced comparable outcomes, reinforcing the effects observed in cardiomyocyte-specific overexpression models [60].

To address whether these findings extend beyond murine models, the effect of PPARα is studied in larger animal models. In a first study, pigs were pretreated with fenofibrate for 4 weeks; however, there was no increase in cardiac *Pparα* or *Cpt1* mRNA levels and fenofibrate did not improve cardiac function after I/R injury [61]. In a second pig study, samples were collected during ischemia and after acute reperfusion. During ischemia, PPARα expression and FAO were maintained, which contrasted with previous reports. However, because mitochondrial respiration and oxidative phosphorylation (OXPHOS) were impaired, this failed to support adequate ATP production and instead promoted lipotoxicity. During acute reperfusion, glucose utilization was increased, indicating activation of glucose metabolism as a compensatory mechanism to restore cardiac energetics, consistent with higher oxygen efficiency of glucose oxidation compared with FAO [62]. In contrast, chronic pharmacological activation of PPARα suppresses glucose metabolism and may therefore limit this metabolic shift, exacerbating energetic imbalance and myocardial injury [63]. Indeed, it is shown that inhibition of FAO can relieve cardiac damage due to upregulation of glucose metabolism [64,65].

Taken together, these findings highlight that metabolic flexibility, rather than sustained FAO, is essential for cardioprotection during I/R injury. The ability to switch toward glucose oxidation represents an important adaptive mechanism under ischemic conditions. In contrast, chronic or excessive activation of PPARα, by maintaining FAO at the expense of glucose utilization, may aggravate energetic imbalance and increase myocardial injury due to limited oxygen availability. In this regard, the timing of PPARα activation appears to be crucial for cardiac outcomes, underscoring that effects are highly context- and time-dependent. Notably, most existing studies have focused on pretreatment strategies, highlighting a lack of data on post-ischemic therapeutic interventions, which may partly explain the contradictory literature.

### 4.2. Upregulated PPARα in Cardiovascular Diseases

Although reduced PPARα signaling is commonly associated with advanced cardiac dysfunction, several pathological conditions are characterized by increased PPARα activity. In particular, metabolic disorders such as obesity and diabetic cardiomyopathy (DCM) are associated with elevated fatty acid availability, which can enhance PPARα signaling and promote fatty acid oxidation pathways. While this response may initially serve as a metabolic adaptation, chronic activation of PPARα can contribute to metabolic inflexibility and lipid accumulation, thereby exacerbating cardiac dysfunction. In the following section, DCM is discussed in greater detail, as it provides a well-characterized context for discussing the PPARα-mediated metabolic remodeling in the heart, in contrast to the more limited recent literature on obesity.

DCM is a diabetes-related disorder of the left ventricle characterized by structural remodeling, including interstitial fibrosis and myocardial hypertrophy, together with functional decline manifested as reduced ejection fraction, occurring independently of traditional cardiovascular risk factors. It is estimated that up to 30% of patients with diabetes exhibit features of DCM, which is correlated with higher mortality [66]. A central feature of DCM is metabolic remodeling, characterized by increased myocardial lipid uptake and elevated FAO, driven in part by upregulation of PPARα. Over time, the balance between lipid uptake and utilization becomes dysregulated, leading to lipid accumulation and lipotoxicity. This contributes to mitochondrial dysfunction, oxidative stress and impaired contractile performance. Furthermore, reduced glucose utilization contributes to metabolic inflexibility, resulting in less efficient energy production and a diminished capacity to respond to stress stimuli. The sustained reliance on FAO also increases oxygen demand and overloads mitochondria with OXPHOS substrates, progressively impairing OXPHOS and exacerbating energetic imbalance in the long term [67,68,69].

To assess whether PPARα upregulation is causally involved in the development of DCM, Finck et al. (2002) compared several established murine models of diabetes with a cardiac-specific PPARα overexpression mouse model [67]. They demonstrated that cardiac PPARα overexpression mimics key metabolic features of DCM, including increased protein expression of several fatty acid metabolism proteins and reduced myocardial glucose transporters. Consistently, in other animal models of diabetes, including diabetic rat models, PPARα expression is similarly upregulated and associated with dysregulated glucolipid metabolism [70]. Furthermore, in a porcine model of early diabetes, differentially expressed genes between control and diabetic animals are enriched for pathways involved in fatty acid metabolism and abnormalities in glucose metabolism, coinciding with myocardial lipotoxicity and early LV diastolic dysfunction [71]. Finally, serum analysis of diabetic patients also reports increased PPARα expression, indicating that this phenomenon is conserved across species [72]. These findings support the concept that excessive cardiac PPARα activation is maladaptive rather than protective due to the loss of metabolic flexibility and lipotoxicity.

Recent research has focused on elucidating the molecular mechanisms underlying PPARα upregulation in DCM. Yin (2019) identified miR-30c as a key regulator in this context. This miRNA, which is downregulated in db/db mice, is associated with increased PGC-1β and PPARα expression [73]. Adenoviral overexpression of miR-30c in cardiomyocytes restored its levels and reduced PGC-1β and PPARα levels, accompanied by enhanced myocardial glucose utilization, reduced lipotoxicity and improved cardiac function. Moreover, another study examined the contribution of super-enhancer-driven noncoding RNAs and identified a PPARα-associated super-enhancer RNA (PPARα-seRNA) as a regulator of cardiac metabolic remodeling. PPARα-seRNA overexpression in a murine DCM model exacerbated metabolic dysfunction, whereas PPARα-seRNA knockdown alleviated metabolic disturbances in vitro. Mechanistically, PPARα-seRNA promoted nuclear translocation of the histone demethylase *Kdm4b*, reducing repressive histone methylation at the PPARα promoter region and thereby enhancing PPARα transcription [74]. Despite these advances, a unifying mechanism explaining the sustained PPARα upregulation in DCM remains to be fully elucidated.

Given the central role of metabolic dysregulation and PPARα overexpression in DCM, therapeutic strategies are increasingly targeting these pathways. Preclinical studies demonstrate that honokiol, a bioactive compound derived from Magnolia Officinalis, activates sirtuin 3 (SIRT3), leading to the deacetylation of metabolic regulators, including PPARα. This modulation is associated with reduced FAO and attenuation of myocardial injury [75]. Similarly, Hmgcs2 knockdown using shRNA showed comparable effects, resulting in decreased myocardial injury and improved cardiac outcomes in models of DCM [76]. In clinical settings, fenofibrate has been reported to improve left ventricular diastolic function in patients with diabetes [77]. Nevertheless, the therapeutic modulation of PPARα requires further investigation to ensure proper metabolic balance and preserve myocardial metabolic flexibility. Additionally, the cardiomyocyte-specific PPARα knockout can be challenged with a DCM model to evaluate protective effects and reinforce previous findings.

### 4.3. Dynamic Regulation of PPARα in Cardiovascular Diseases

In septic cardiomyopathy (SICM), PPARα signaling is dynamically regulated throughout disease progression. The condition is characterized by an initial adaptive phase in which PPARα activity is enhanced to support cardiac energy demands, followed by progressive downregulation during maladaptive remodeling and heart failure. This time-dependent regulation adds complexity to therapeutic strategies targeting PPARα, as interventions may have stage-specific effects depending on disease progression. In the section below, SICM is discussed in greater detail.

Sepsis is a life-threatening inflammatory syndrome resulting from a dysregulated host response to infection, accounting for 1 out of 5 deaths worldwide [78]. Besides the inflammatory point of view, sepsis is increasingly recognized as a metabolic disease marked by mitochondrial dysfunction and impaired energy metabolism. Organ failure is the defining clinical consequence of sepsis, and with the heart among the most affected organs, this is closely correlated with poor outcomes. The reported prevalence of SICM varies widely, ranging from approximately 20% in recent meta-analyses to 10-70% in other studies, largely due to the lack of a uniform definition [79,80]. Nevertheless, SICM is consistently associated with increased mortality in septic patients [79,80]. It is mostly described as a reversible condition of cardiac dysfunction characterized by acute left ventricular impairment with reduced contractility and ejection fraction. In addition, cardiac metabolism is altered during sepsis. In experimental models resembling the early, hyperdynamic stage of sepsis, cardiac FAO is transiently augmented to meet the increased energetic demands required to sustain cardiac performance. This is accompanied by enhanced systolic function, increased fractional shortening and elevated myocardial oxygen consumption. Notably, a similar hyperdynamic phenotype has been described in septic patients, in whom elevated left ventricular systolic function is observed during the early phase of sepsis [81]. However, PPARα levels were not measured in this hyperdynamic stage. After this early hyperdynamic stage, PPARα expression is markedly decreased, which is associated with reduced myocardial FAO [82]. A similar decline in PPARα activity and FAO capacity has also been observed in the liver during sepsis [83].

To investigate the role of PPARα in cardiac functioning during sepsis, whole-body PPARα-deficient mice were generated and subjected to cecal ligation and puncture (CLP), a model resembling peritoneal sepsis. Following CLP, PPARα-deficient mice exhibited significantly reduced survival outcomes compared to wild-type controls, accompanied by higher tissue bacterial load [84]. In addition, the PPARα-deficient mice have increased circulating cardiac troponin levels, indicative of myocardial injury. Histological analysis of left ventricular tissue revealed structural abnormalities, such as swollen cardiomyocytes, cytoplasmic fragmentation and loss of nuclei, pointing to cellular degradation and necrosis. At the metabolic level, full-body PPARα-deficiency resulted in downregulation of genes involved in FAO in the heart and lower blood glucose levels 24 h after CLP compared with wild-type mice [85]. Collectively, these findings indicate that intact PPARα signaling is critical for maintaining cardiac metabolic and structural homeostasis and improving survival during sepsis.

Although studies using global PPARα-deficient mice demonstrated the importance of PPARα in cardiac injury during sepsis, these models cannot distinguish between systemic and cardiomyocyte-specific mechanisms. To address this limitation, a cardiomyocyte-specific inducible knockout model is studied in the sepsis context. This study largely confirmed the phenotype observed in whole-body knockout animals. Following lipopolysaccharide (LPS) administration, cardiomyocyte-specific PPARα-deficient mice exhibited worse cardiac function, with reduced ejection fraction and fractional shortening measured by echocardiography, compared to wild-type mice. In addition, higher infiltration of inflammatory cells is seen in the myocardium of knockout mice compared to control subjects. Mitochondrial injury was exacerbated in knockout hearts, as demonstrated by transmission electron microscopy. This was accompanied by a decreased mitochondrial respiratory complex activity, resulting in increased ROS. Metabolically, fatty acid metabolism was suppressed, while mitophagy was enhanced. So far, no studies exist with a cardiac-specific knockout model tested in a polymicrobial sepsis model [86].

As mentioned before, PPARα has been implicated in regulating macrophage polarization. PPARα promotes polarization towards an anti-inflammatory phenotype, thereby limiting excessive inflammatory responses. Mechanistically, this effect is mediated through the regulation of DUSP1, a key negative regulator of MAPK signaling. In vitro stimulation of macrophages with LPS reduced PPARα expression, which in turn decreased DUSP1 levels and permitted sustained activation of MAPK pathways. Consequently, macrophages shifted toward a pro-inflammatory phenotype with increased cytokine production. Moreover, coculture of LPS-stimulated macrophages with cardiomyocytes led to increased cardiac injury markers such as cardiac troponin and CK-MB. Conversely, overexpression of PPARα in macrophages restored DUSP1 signaling and reduced myocardial injury markers in cocultured cardiomyocytes, supporting a DUSP1-dependent immunomodulatory role of PPARα in vitro [87]. In vivo, myeloid-specific deletion of PPARα did not significantly alter cardiac injury or myocardial immune responses, although the specific PPARα-DUSP1 interaction was not investigated in these studies [86]. Together, these findings suggest that although the PPARα-DUSP1 axis regulates macrophage inflammatory signaling under controlled conditions, the cardioprotective effects of PPARα during sepsis are predominantly driven by its cardiometabolic functions rather than immune-cell-specific anti-inflammatory signaling. This discrepancy likely reflects the systemic nature of sepsis in vivo, where whole-organism metabolic and inflammatory interactions may buffer or compensate for immune-cell-specific PPARα effects observed in isolated in vitro systems.

Recent advances in the pathophysiology of SICM have highlighted a central role of PPARα, making its agonists potential therapeutics. The PPARα activator WY-14643 alleviated cardiac injury and mitochondrial dysfunction in LPS-treated models, but was given prophylactically, leaving its therapeutic potential untested [86]. Fenofibrate similarly improved cardiac contractility and reduced metabolic and inflammatory disturbances in LPS- and CLP-pretreated animals [87,88,89]. In humans, pre-LPS administration of fenofibrate did not lower cytokines or acute-phase proteins [90]. However, further studies with PPARα ligands are needed across in vivo sepsis models and in patients to confirm their clinical potential. Novel therapeutic strategies increasingly focus on mechanisms linked to PPARα signaling. One approach involves 4-phenylbutyric acid (PBA), a compound known to modulate multiple pathways, including inhibition of endoplasmic reticulum (ER) stress, histone deacetylase activity, and mitochondrial biogenesis, and lead to PPARα modulation. In a CLP rat model, pretreatment with PBA improved survival and reversed the PPARα reduction seen in septic hearts. This cardioprotective effect was associated with modulation of metabolic and inflammatory pathways, including improved amino acid and lipid metabolism, potentially mediated through regulation of *Pparα*, *Comt* and *Ptgs2* [91].

Another emerging target in SICM is ferroptosis. PPARα appears closely linked to ferroptosis: PPARα knockout mice exhibit enhanced ferroptosis, while induction of ferroptosis suppresses PPARα expression, suggesting a two-way interaction [92,93]. In a recent in vivo sepsis study, myocardial lipidomic profiling revealed that 14,15-EET, an arachidonic acid-derived lipid mediator and ferroptosis inhibitor, was significantly downregulated. Pre-treatment with TPPU, a soluble epoxide hydrolase inhibitor that prevents 14,15-EET degradation, improved cardiac and mitochondrial dysfunction in CLP mice. These effects were associated with reduced ferroptosis and restored PPARα expression, highlighting ferroptosis inhibition as a promising therapeutic strategy in sepsis-induced cardiomyopathy. Notably, pharmacological inhibition of PPARα with GW6471 eliminated all cardioprotective effects, indicating that PPARα acts downstream of 14,15-EET [94].

Collectively, current evidence indicates that PPARα is a key determinant of cardiac adaptation during sepsis, primarily by preserving mitochondrial function and metabolic flexibility rather than solely modulating inflammation. Loss of PPARα signaling induces energy failure and contractile dysfunction, whereas its restoration consistently improves cardiac performance in preclinical models. Nonetheless, therapeutic targeting of PPARα requires further validation in human studies. Moreover, potential systemic side effects need to be carefully validated, as heart-specific delivery of PPARα activators or related compounds remains a significant challenge.

## 5. Challenges and Future Perspectives for PPARα Modulation in Cardiovascular Diseases

Since PPARα is dysregulated in several cardiovascular diseases, as described above, it represents an attractive therapeutic target. Numerous preclinical studies demonstrated that pharmacological intervention or genetic modulation of PPARα improves key metabolic parameters, including mitochondrial function, fatty-acid oxidation and oxidative stress, while also limiting inflammation across cardiovascular disease models such as I/R injury and SICM [57,86,87]. Together, these studies demonstrate that PPARα plays a central role in maintaining cardiac energy homeostasis under stress.

In experimental research, the synthetic PPARα agonists WY-14643 and GW7647 are frequently used. Short-term administration in animal models with SICM consistently improves cardiac metabolic flexibility and limits myocardial injury, making this a valuable pharmacological tool [58,86]. However, chronic exposure shows significant toxicity, including oxidative DNA damage, hepatocyte proliferation and liver tumor formation in rodents within approximately 5 months, which limits clinical translation [95]. To address these concerns, milder PPARα agonists such as fibrates have been developed. Although these agents, including fenofibrate, only provide partial PPARα activation, they demonstrate beneficial metabolic and anti-inflammatory effects in preclinical cardiovascular disease models [88,89,96].

### 5.1. Clinical Trials with Fibrates

Until now, fibrates have mainly been evaluated in large clinical trials aimed at reducing the cardiovascular risk in patients with type 2 diabetes. In the FIELD study (fenofibrate intervention and event lowering in diabetes), fenofibrate treatment resulted in a modest reduction in nonfatal myocardial infarction; however, strong cardioprotective effects as observed in preclinical models were not translated into clinical outcomes [97]. Similarly, the ACCORD trial (action to control cardiovascular risk in diabetes) investigated the additional benefits of fibrates combined with statin therapy, but no overall reduction in cardiovascular events was observed [98]. More recently, the PROMINENT trial (pemafibrate to reduce cardiovascular outcomes by reducing triglycerides in patients with diabetes) evaluated pemafibrate in diabetic patients and demonstrated significant triglyceride lowering but without improvement in cardiovascular outcomes [99].

Additionally, more studies have explored fibrate treatment in diabetic patients with developed heart failure. Fenofibrate improved lipid parameters and reduced rehospitalization six months after treatment, while other studies reported modest beneficial effects in patients with both heart failure and type 2 diabetes [100,101]. Nevertheless, no major clinical trials have yet investigated the therapeutic potential of PPARα modulation on I/R or SICM.

Currently, a clinical trial is evaluating fenofibrate treatment in patients with type 2 diabetes to assess its effects on cardiovascular events (NCT05542147). In contrast to previous studies, this trial will focus on the underlying genetic heterogeneity in the PPARα gene that may influence the cardiovascular response to fenofibrate therapy. To our knowledge, no other large clinical trials with PPARα modulation are planned.

### 5.2. Challenges for PPARα Modulation in Cardiovascular Diseases

Although PPARα has emerged as an important regulator of cardiac metabolism and homeostasis, translating experimental findings into effective clinical therapies remains challenging. In the following sections, the major challenges and limitations of targeting PPARα in cardiovascular diseases are discussed.

#### 5.2.1. Disease- and Stage-Dependent Effects

An underlying mechanism for explaining the gap between preclinical and clinical results is that cardiovascular diseases should not be understood simply as a uniform reduction or increase in PPARα signaling; they also involve a loss of metabolic flexibility. Healthy hearts dynamically switch energy production between fatty acids, glucose and other alternative substrates depending on energetic demand and substrate availability. In diseases like SICM, this cardiac metabolic flexibility is lost, and metabolism becomes locked into maladaptive patterns.

Consequently, PPARα activation must be carefully timed, as it may be beneficial during acute energetic stress but become detrimental during chronic metabolic overload. In I/R, activation of PPARα during ischemia may exacerbate injury due to limited oxygen availability for fatty acid oxidation, whereas activation during reperfusion can support metabolic recovery by restoring energy production and promoting cardiac repair. Another example, SICM initially presents with a hyperdynamic state, where FAO is activated, followed by a phase of suppression, where PPARα activation can be beneficial. This phase-dependent behavior likely contributes to the discrepancy between uniform benefits in controlled preclinical models and heterogeneous responses in patients.

#### 5.2.2. Limited Translation from Animal Models

One of the major challenges in translating preclinical findings on PPARα modulation into clinical therapies is patient heterogeneity. In vitro or in vivo models are relatively controlled systems with limited biological variability, whereas patients with cardiovascular diseases represent a highly heterogeneous population in terms of genetics, comorbidities, medication use and environmental influences. In addition, cardiovascular diseases such as heart failure are a heterogeneous group of pathologies with different origins, leading to slightly different pathological mechanisms. Moreover, cardiovascular diseases such as heart failure or cardiac hypertrophy develop progressively over time, involving dynamic compensatory and maladaptive remodeling processes that are often not fully recapitulated in experimental models.

This complexity is further increased by the fact that the same clinical diagnosis can encompass distinct phenotypes with different underlying metabolic profiles and disease trajectories. As a result, therapeutic responses to PPARα modulation may vary substantially between patient subgroups. Therefore, improved patient stratification based on metabolic, genetic or phenotypic characteristics is likely essential to identify subpopulations that may benefit from PPARα-target therapies. This approach is increasingly recognized in recent clinical trial designs (NCT05542147), where subgroup analysis has suggested that specific metabolic/genetic phenotypes may respond more favorably to fibrate treatment, potentially masking beneficial effects in unstratified populations.

#### 5.2.3. Selectivity of PPARα Modulation

Currently, fibrates are administered systemically in clinical trials, which means that PPARA activation occurs across multiple organs rather than restricted to the heart. This can lead to off-target effects in tissues such as the liver, where PPARα activation may induce organ-specific toxicity or unwanted metabolic disturbances.

In addition, the heart itself is a highly heterogeneous organ composed of cardiomyocytes, endothelial cells, fibroblasts and immune cells, each of which responds differently to PPARα modulation. In cardiomyocytes, PPARα primarily regulates FAO and ATP production, whereas in immune and endothelial cells, it is more strongly involved in inflammatory signaling pathways. As a result, systemic pharmacological activation may generate cell-type-specific effects that are not always synergistic, potentially leading to competing biological responses that blunt overall therapeutic efficacy and help explain the limited translation of preclinical success to clinical outcomes.

As an example, in SICM, combined metabolic and inflammatory dysregulation is present. In this context, simultaneous activation of fatty acid oxidation in cardiomyocytes and anti-inflammatory pathways in immune cells could be beneficial for restoring energy balance and mitigating cytokine-driven injury. However, if immune-suppression pathways become too strong, this can impair pathogen clearance and worsen infection control.

These observations highlight the need for organ- or cell-specific therapeutic strategies. Cardiac-specific delivery, combined with careful titration of receptor activation, could minimize off-target effects in other organs. One potential strategy is cardiac gene transfer using adeno-associated viral (AAV) vectors in conditions characterized by reduced myocardial PPARα expression, such as SICM. Certain AAV serotypes, for example AAV9, preferentially target cardiomyocytes, enabling relatively selective cardiac delivery. Another complementary strategy is to identify and therapeutically modulate upstream regulators of PPARα signaling within cardiomyocytes themselves rather than directly activating the receptor in several cell types. Nevertheless, cardiovascular diseases remain complex disorders involving multiple dysregulated pathways. Therefore, future research should focus on combination therapies that simultaneously target several affected mechanisms rather than a single molecular pathway, such as approaches that concurrently modulate metabolism and immune cell infiltration (particularly for SICM), combine oxidative stress reduction with PPARα activation, or target pathways that preserve mitochondrial function.

## 6. Conclusions

PPARα plays a central role in cardiac metabolism and function, and extensive research has demonstrated its involvement in a wide range of cardiovascular pathologies. Dysregulation of PPARα occurs in a disease-specific manner, including upregulation in diabetic cardiomyopathy, downregulation in ischemia and reperfusion injury, cardiac hypertrophy and heart failure, as well as dynamic regulation during septic cardiomyopathy. These alterations are associated with distinct metabolic and functional consequences that contribute to disease progression. Consequently, modulation of PPARα signaling remains an attractive therapeutic strategy across multiple cardiovascular diseases. Nevertheless, several challenges still need to be addressed, including cell-type specificity, time- and disease-stage-dependent modulation, and improved patient stratification to account for clinical heterogeneity. Despite these limitations, continued advances in understanding PPARα biology may pave the way for more precise and effective therapeutic interventions in cardiovascular diseases.

## Figures and Tables

**Table 1:** Overview of recent literature on heart failure showing the protective effects of PPARα signaling.

Study	Condition	Study Type	Model	Description
Briand, 2021 [35]	HFpEF	Therapeutic intervention	In vivo hamster model	The PPARα/δ agonist elafibranor was evaluated in a model of NASH/HFpEF. Hamsters treated with elafibranor had significant improvement in diastolic dysfunction and filling pressure.
Han, 2021 [36]	Chronic heart failure	Mechanism of therapeutic	In vivo rat model	Yiqi Fumai lyophilized injection (YQFM) is used as a treatment for chronic heart failure. YQFM is upregulating several proteins involved in mitochondrial function and OXPHOS, including PPARα. In addition, cardiomyocyte apoptosis is reduced.
Zhang, 2022 [37]	Chronic heart failure	Therapeutic intervention	In vivo rabbit model	Cardiac contractility modulation is applied to rabbits with heart failure. This ameliorated several aspects of heart failure, including cardiac structure and function. In addition, cardiac metabolism is restored with higher protein levels of PPARα and AMPK.
Sunagawa, 2022 [38]	Heart failure	Therapeutic intervention	In vitro cultured cardiomyocytes from neonatal rats and an in vivo rat model	Auraptene, a citrus-peel-derived natural product, was evaluated in in vitro and in vivo models of heart failure. It activated PPARα-dependent expression in vitro, and improved systolic function and fibrosis in rats, accompanied by upregulation of PPARα target genes.
Li, 2023 [39]	Heart failure	Mechanistic study/therapeutic intervention	In vivo rat model and in vitro neonatal rat cardiomyocytes	Heart failure increases Fas-associated death domain (FADD) expression, promoting its interaction with PPARα and thereby inhibiting PPARα activity. GRb1, a natural product derived from ginseng, disrupted FADD upregulation and prevents PPARα-FADD binding, leading to improved cardiac function, increased ATP production and restoration of metabolic homeostasis.
Yang, 2024 [40]	Heart failure	Mechanistic study	In silico predictions In vivo mouse model	The mechanisms of Shenmai injection (SMI), a Chinese medicine formulation, are investigated in heart failure. In silico analysis identified PPARα as a key target, and active SMI components showed high binding affinity to PPARα. This was associated with improved cardiac function, decreased inflammatory cell infiltration and better mitochondrial function.
Wang, 2024 [41]	Heart failure	Mechanistic study	In vitro AV16 and human cardiac microvascular endothelial cells	PPARα is part of a cardioprotective signaling axis involving SIRT1 and NCOR1. Overexpression of SIRT1 increases PPARα activity, which in turn enhances NCOR1 transcription. This pathway leads to reduced ROS production and lipotoxicity, improved mitochondrial metabolism and ATP production, decreased inflammation and apoptosis.

**Table 2:** Recent literature on cardiac hypertrophy and PPARα signaling, where modulation of PPARα shows protective effects.

Study	Condition	Study Type	Model	Summary
Zhang, 2021 [42]	Cardiac hypertrophy	Therapeutic intervention	In vivo rat model	Bawei Chenxiang Wan (BCW), a Chinese medicine formulation, improved cardiac function and structural remodeling during hypertrophy. These effects were associated with metabolic normalization via upregulation of PPARα and AMPK signaling.
Kumari, 2022 [43]	Cardiac hypertrophy	Mechanistic study	In vivo mouse model	PPARα-null mice subjected to cardiac hypertrophy exhibited altered cardiac proteomic profiles, including reduced apoptotic markers and increased autophagy-related proteins. This suggests that PPARα may regulate cardiomyocyte apoptosis and modulate autophagic processes during hypertrophic remodeling.
Liu, 2022 [44]	Cardiac hypertrophy	Mechanistic study	In vitro cardiomyocytes and an in vivo mouse model	Long non-coding RNA MHRT protects against cardiac hypertrophy by promoting SIRT1 SUMOylation, which activates PPARα signaling. This enhances mitochondrial function and fatty acid oxidation, thereby attenuating hypertrophic remodeling
Gao, 2022 [45]	Cardiac hypertrophy	Therapeutic intervention	In vitro H9C2 cell model	Salidroside attenuated hypertrophic markers while increasing PPARα expression. This effect was mediated by ATGL, an upstream activator of PPARα.
Zhu, 2021 [27]	Pressure overload cardiac hypertrophy	Mechanistic study	In vivo PPARα-MHC-deficient mouse model	Mice with a cardiomyocyte-specific deletion of PPARα developed accelerated cardiac hypertrophy and increased fibrosis. This indicates that PPARα plays a protective role in hypertrophy by regulating fatty acid oxidation and maintaining extracellular matrix homeostasis.
Bianchi, 2025 [46]	Cardiac hypertrophy	Mechanistic study	In vitro cardiomyoblast	Silencing of PPARα led to the induction of a hypertrophic phenotype, with increased NPPB, mitochondrial dysfunction and impaired lipid metabolism.
Wang, 2025 [47]	Cardiac hypertrophy	Therapeutic intervention	In vitro cardiomyocyte and in vivo rat model	Transfer RNA-derived small RNAs may have an important role in the development of cardiac hypertrophy. tRF-16-R29P4PE was identified as significantly downregulated in patients with cardiac hypertrophy. Modulation of tRF-16-R29P4PE led to reduced hypertrophic markers and induction of PPARα.
Zhao, 2025 [48]	Pressure overload-induced hypertrophy	Mechanistic study	In vivo mouse model and in vitro cardiomyocytes	*Lgr6* is a regulator of cardiac hypertrophy. *Lgr6* is downregulated in hypertrophy, together with PPARα. *Lgr6* overexpression attenuated cardiac hypertrophy and dysfunction by upregulating the expression of PPARα, thereby promoting metabolic reprogramming in cardiomyocytes.
Xuan, 2025 [49]	Basal	Mechanistic study	In vitro neonatal rat cardiomyocytes	PPARα expression was modulated in cardiomyocytes using pharmacological activators and inhibitors, with hypertrophic markers as readout. Activation by Wy-14643 promoted cardiomyocyte proliferation and reduced hypertrophy, whereas inhibition induced hypertrophic changes and suppressed proliferation. These findings indicate a protective role for PPARα against hypertrophy.
Wheeler, 2026 [50]	Cardiac hypertrophy	Mechanistic study	In vitro cardiomyocytes and an in vivo mouse model	*Redd1* is upregulated upon cardiac hypertrophy and is associated with reduced PPARα expression and a decrease in fatty acid oxidation gene levels. Deletion of *Redd1* restores PPARα signaling and cardiac metabolic function, suggesting a potential therapeutic target for hypertrophy.

## Data Availability

No new data were created or analyzed in this study.
